# Adolescents With ADHD Do Not Take Longer to Recover From Concussion

**DOI:** 10.3389/fped.2020.606879

**Published:** 2021-01-13

**Authors:** Nathan E. Cook, Grant L. Iverson, Bruce Maxwell, Ross Zafonte, Paul D. Berkner

**Affiliations:** ^1^Department of Physical Medicine and Rehabilitation, Harvard Medical School, Boston, MA, United States; ^2^Mass General Hospital for Children Sports Concussion Program, Boston, MA, United States; ^3^Spaulding Rehabilitation Hospital, Charlestown, MA, United States; ^4^Discovery Center for Brain Injury and Concussion Recovery, Spaulding Research Institute, Charlestown, MA, United States; ^5^Department of Computer Science, Colby College, Waterville, ME, United States; ^6^Department of Physical Medicine and Rehabilitation, Brigham and Women's Hospital, Boston, MA, United States; ^7^College of Osteopathic Medicine, University of New England, Biddeford, ME, United States

**Keywords:** attention deficit hyperactivity disorder (ADHD), mild traumatic brain injury, injury surveillance, brain trauma, outcome research, prognosis

## Abstract

The objective of this study was to determine whether adolescents with attention-deficit/hyperactivity disorder (ADHD) have prolonged return to school and sports following concussion compared to those without ADHD and whether medication status or concussion history is associated with recovery time. We hypothesized that having ADHD would not be associated with longer recovery time. This prospective observational cohort study, conducted between 2014 and 2019, examined concussion recovery among school sponsored athletics throughout Maine, USA. The sample included 623 adolescents, aged 14–19 years (mean = 16.3, standard deviation = 1.3 years), 43.8% girls, and 90 (14.4%) reported having ADHD. Concussions were identified by certified athletic trainers. We computed days to return to school (full time without accommodations) and days to return to sports (completed return to play protocol) following concussion. Adolescents with ADHD [median days = 7, interquartile range (IQR) = 3–13, range = 0–45] did not take longer than those without ADHD (median days = 7, IQR = 3–13, range = 0–231) to return to school (*U* = 22,642.0, *p* = 0.81, *r* = 0.01; log rank: $\chi_{1}^{2}$ = 0.059, *p* = 0.81). Adolescents with ADHD (median days = 14, IQR = 10–20, range = 2–80) did not take longer than those without ADHD (median days = 15, IQR = 10–21, range = 1–210) to return to sports (*U* = 20,295.0, *p* = 0.38, *r* = 0.04; log rank: $\chi_{1}^{2}$ = 0.511, *p* = 0.48). Medication status and concussion history were not associated with longer recovery times. Adolescents with ADHD did not take longer to functionally recover following concussion. Recovery times did not differ based on whether adolescents with ADHD reported taking medication to treat their ADHD or whether they reported a prior history of concussion.

## Introduction

A concussion is a mild traumatic brain injury (1). Primary care pediatrics is the most common initial point of concussion care for children (2). In a recent survey of pediatricians, essentially all (99%) had treated at least one patient, and half (50%) had treated six or more patients for concussion in the previous year (3). Professional consensus statements have identified attention-deficit/hyperactivity disorder (ADHD), a common neurodevelopmental disorder marked by problems with inattention, difficulty concentrating, poor impulse control, and excessive activity (4), as an important preexisting health condition to consider with regard to concussion management and recovery (5–7). Youth with ADHD are at greater risk of bodily injuries (8–11) and have a greater lifetime history of concussions (12–16) compared to those without ADHD. Pediatricians also play a central role in treating and managing ADHD. Almost half of youth with ADHD (42%) receive treatment for their symptoms solely through their primary care physician (17).

Most concussed children and adolescents experience rapid symptom improvement during the first 2 weeks after injury, and symptoms lasting longer than 1 month are considered “persistent” (1). Although studies of more severe forms of neurotrauma suggest that ADHD might be associated with worse outcome (18–20), the role of ADHD as a risk factor for prolonged symptoms or worse outcome following sport-related concussion remains poorly understood. A systematic review examining predictors of clinical recovery from concussion concluded that available studies do not support an association between ADHD and worse clinical outcome (21). A follow-up systematic review focused specifically on the role of preexisting ADHD as a risk factor for prolonged symptoms or worse outcome following concussion identified several major limitations regarding the available literature that preclude definitive conclusions (22). For example, only one study to date has been specifically designed to examine if ADHD was associated with prolonged concussion recovery and that study did not find an association (23). Moreover, two-thirds of the studies included fewer than 15 ADHD cases, no studies examined time to return to school as an outcome, no studies examined whether youth with ADHD who are taking medication experience different recovery trajectories, and no studies considered or controlled for prior concussions as an important potential covariate (22). Using a large, injury surveillance database, the current study sought to prospectively examine whether adolescents with ADHD have prolonged return to school and sports following concussion compared to those without ADHD, and whether medication status and concussion history are associated with recovery time. We hypothesized that having ADHD would not be associated with longer recovery time.

## Materials and Methods

### Participants

This is a prospective observational cohort study of adolescent student athletes throughout the state of Maine, USA, conducted between 2014 and 2019 (5 academic years). Athletic trainers or another school official used the Head Injury Tracker (HIT), a free injury surveillance application created by the Maine Concussion Management Initiative, to monitor concussion recovery. In the first year, the HIT was deployed to 4 schools, 28 schools were enrolled during the second year, and by the final year, it was used in 10 schools. The HIT database includes 673 adolescents aged 14–19 years who sustained a sport-related concussion between September 2014 and June 2019. From this original sample, 30 were excluded because dates were missing for both return to school and return to sports, and 20 were excluded because of problematic dates that, because of data deidentification procedures, could not be confirmed/checked for accuracy and had a high probability of being data entry errors.

The final sample included 623 adolescents aged 14–19 years (mean = 16.3, standard deviation = 1.3 years). The sample was almost equally split between girls (*n* = 273, 43.8%) and boys (*n* = 350, 56.2%). Of these athletes, 90 (14.4%) self-reported a preinjury history of ADHD. A higher proportion of boys reported having preinjury ADHD compared to girls (19.7 vs. 7.7%, χ^2^ = 17.94, *p* < 0.001). For boys, the sports played at the time of injury were football (36.9%), soccer (20%), basketball (9.1%), lacrosse (8.6%), ice hockey (6.9%), and several other sports (18.5%). For girls, the sports played at the time of injury were soccer (34.8%), basketball (12.1%), spirit squad (12.1%), field hockey (8.1%), ice hockey (5.1%), and several other sports (27.8%). Among the students with ADHD, 37 (41.1%), self-reported taking medication to treat their ADHD.

Date of return to school was not available for 12 participants (1.9%), and date of return to sports was not available for 35 participants (5.6%). Rates of missing outcome data did not differ between sexes (missing school return data: boys = 2.3%, girls = 1.5%, $\chi_{1}^{2}$ = 0.55, *p* = 0.46; missing sports return data: boys = 5.7%, girls = 5.5%, $\chi_{1}^{2}$ = 0.01, *p* = 0.91), and there were no differences in rates of missing outcome data based on ADHD status (missing school return data: ADHD = 2.2%, no ADHD = 1.9%, $\chi_{1}^{2}$ = 0.05, *p* = 0.83; missing sports return data: ADHD = 4.4%, no ADHD = 5.8%, $\chi_{1}^{2}$ = 0.27, *p* = 0.60).

### Measures

#### Demographic, Health History, and Injury Information

Demographic and self-reported health history information was collected from the adolescents. Injury information collected by an athletic trainer or other school official included the concussion date and the scenario in which the concussion occurred (e.g., team vs. not team activities).

#### Recovery Time

Athletic trainers or other school officials entered the date students returned to school (full time without accommodations) and the date they returned to sports (completed return to play protocol) following their concussion. Recovery time was calculated as the number of days between the injury date and the date of return to school and the date of return to sports.

### Procedures

The HIT application is an online injury surveillance platform. When an adolescent sustained a concussion, an athletic trainer or school official entered information about the injury and their recovery via smartphone or webpage. All athletic trainers/school officials involved in data collection completed an online training about the HIT application format and the process for entering information. Adolescents and schools were not compensated for their participation. A dedicated HIT project coordinator provided technical assistance and data collection oversight. Institutional review board approval for the creation of the deidentified database and its use for research was obtained.

### Statistical Analyses

The outcome variables (days to return to school and days to return to sports) were non–normally distributed; thus, Mann-Whitney *U* tests were employed to assess whether adolescents with ADHD took longer to return compared to those without ADHD. Survival analysis (Kaplan–Meier with log rank tests) was used to compare the recovery times of adolescents with and without ADHD (censored at 28 days). To maximize the clinical relevance of these findings, each adolescent's return to school/sports status was also dichotomized (i.e., returned or not) at various recovery benchmarks (e.g., 1 week, 10 days, 2 weeks, etc.) and χ^2^ tests compared the proportion of those with and without ADHD who had not yet returned to school or sports at these various time points. Cumulative recovery curves were constructed to visually display the proportion of adolescents who returned to school or sports over time. The Mann–Whitney *U*-test *Z*-values were used to calculate a non-parametric effect size *r*, where *r*=$\frac{Z}{\surd N}$ (24), which was interpreted according to conventional guidelines, i.e., *r* = 0.1 (small), *r* = 0.3 (medium), and *r* = 0.5 (large) (25). For χ^2^ tests, odds ratios (ORs) were computed as effect sizes (26) and interpreted according to widely used criteria (27), i.e., ORs between 1.2 and 1.71 (small), ORs between 1.72 and 2.4 (medium), and ORs >2.4 (large). All statistical analyses were conducted using IBM SPSS Statistics 25.

## Results

### Descriptive Data

Demographic characteristics of the sample are presented in Table 1, and descriptive statistics for days to return to school and sports are presented in Table 2. Students' age was not associated with days to return to school (Spearman ρ = −0.06, *p* = 0.14) or days to return to sports (ρ = 0.01, *p* = 0.86). Days to return to school was significantly positively correlated with days to return to sports (ρ = 0.63, *p* < 0.001). The ADHD group contained a significantly greater proportion of boys (76.7%) compared to the no-ADHD group (52.7%), $\chi_{1}^{2}$ = 17.94, *p* < 0.001. However, among the full sample, girls and boys did not differ on days to return to school (*U* = 43,193.0, *p* = 0.19) or sports (*U* = 41,644.5, *p* = 0.65). Youth with ADHD (41.1%) were more likely than those without ADHD (30.4%) to report having a prior concussion, $\chi_{1}^{2}$ = 4.07, *p* = 0.04. A greater proportion of youth with ADHD (18.9%) self-reported a history of depression compared to youth without ADHD (6.4%), $\chi_{1}^{2}$ = 16.03, *p* < 0.001. Among youth reporting a history of depression, rates of medication to treat depression were similar between youth with ADHD (64.7% reporting medication) and without ADHD (61.8% reporting medication).

**Table 1:** Summary of demographic and health history information between groups with ADHD and without ADHD.

	**Total sample**	**ADHD**	**No ADHD**
	**(*N* = 623)**	**(*n* = 90)**	**(*n* = 533)**
Age, mean (SD), years	16.3 (1.3)	16.4 (1.3)	16.3 (1.3)
Female gender, *n* (%)	273 (43.8)	21 (23.3)	252 (47.3)
Number of prior concussions, mean (SD)	0.5 (0.9)	0.7 (1.0)	0.5 (0.9)
Zero prior concussions (*n*, %)	424 (68.1)	53 (58.9)	371 (69.6)
1 prior concussion, *n* (%)	122 (19.6)	23 (25.6)	99 (18.6)
2 prior concussions, *n* (%)	51 (8.2)	9 (10.0)	42 (7.9)
3 or more prior concussion, *n* (%)	26 (4.3)	5 (5.5)	21 (3.9)
Migraine history, *n* (%)	71 (11.4)	13 (14.4)	58 (10.9)
Depression history, *n* (%)	51 (8.2)	17 (18.9)	34 (6.4)
Depression medication, *n* (%)	32 (5.1)	11 (12.2)	21 (3.9)

*ADHD, attention-deficit/hyperactivity disorder*.

**Table 2:** Number of days to return to school and sports by ADHD status, gender, and prior concussion subgroups.

	**Days to return to school**							**Days to return to sports**						
	***n***	**Mean**	**Median**	**SD**	**IQR**	**Range**	**Days until 90% return**	***n***	**Mean**	**Median**	**SD**	**IQR**	**Range**	**Days until 90% return**
Total sample	611	11.0	7	17.2	3–13	0–231	23	588	19.6	14.5	21.5	10–21	1–210	34
No ADHD	523	11.2	7	18.2	3–13	0–231	23	502	19.9	15	22.7	10–21	1–210	34
ADHD	88	9.5	7	9.3	3–13	0–45	21	86	17.5	14	12.9	10–20	2–80	34
ADHD, medication	36	10.1	8	9.5	3–12	0–45	21	34	18.5	14	12.8	9–22.75	5–53	42
ADHD, no medication	52	9.2	6	9.2	3–13.75	0–41	17	52	16.9	14	13.1	10–18	2–80	28
Boys	342	10.9	7	19.1	3–12.25	0–231	21	330	19.3	14	20.1	10–21	2–210	34
Girls	269	11.1	7	14.6	3–14	0–177	25	258	20.0	15	23.3	10–21	1–207	35
No ADHD boys	275	11.3	7	20.8	3–12	0–231	21	264	19.7	14	21.5	11–21	2–210	34
ADHD boys	67	9.5	7	9.2	3–13	0–41	25	66	17.4	14	13.4	9–21	2–80	34
No ADHD girls	248	11.2	7	14.9	3–14	0–177	26	238	20.2	15	24.0	10–21.25	1–207	35
ADHD girls	21	9.7	7	9.8	4–12.5	1–45	17	20	17.9	16	11.6	11–18	5–53	21
ADHD prior concussion	36	7.5	6.5	5.6	3–11	0–21	16	35	16.4	13	13.4	10–20	5–80	22
ADHD no prior concussion	52	10.9	7	11.0	3.25–14	0–45	29	51	18.3	14	12.7	10–25	2–56	35
No ADHD prior concussion	160	12.7	6	26.7	3–13	0–231	21	147	22.6	15	29.5	10–24	3–207	35
No ADHD no prior concussion	363	10.6	7	12.8	3–14	0–131	24	355	18.8	15	19.1	10–21	1–210	34

*ADHD, attention-deficit/hyperactivity disorder; SD, standard deviation; IQR, interquartile range*.

### Return to School

**Figure 1:**
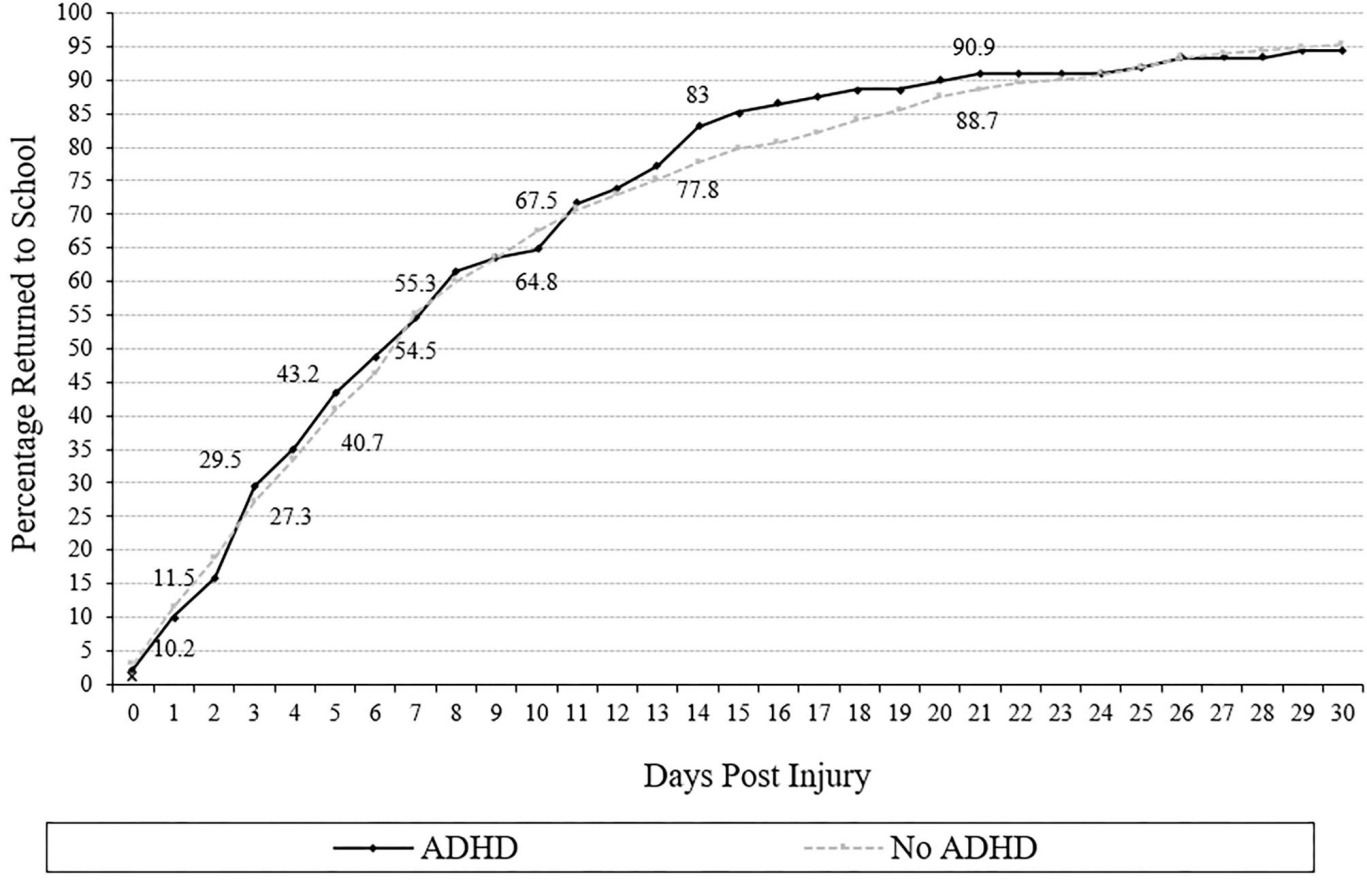
Days to return to school (cumulative percentage curves). Cumulative percentage curves by group of time to return to school. The featured percentages are as follows: 1 day (10.2% of those with ADHD, 11.5% of those without ADHD), 3 days (29.5% of those with ADHD, 27.3% of those without ADHD), 5 days (43.2% of those with ADHD, 40. 7% of those without ADHD) 7 days (54.5% of those with ADHD, 55.3% of those without), 10 days (64.8% of those with ADHD, 67.5% of those without ADHD), 14 days (83.0% of those. with ADHD, 77.8% of those without), and 21 days post injury (90.9% of those with ADHD, 88. 7% of those without ADHD).

Cumulative recovery curves displaying the proportion of adolescents with and without ADHD who returned to school over time are presented in Figure 1. Adolescents with ADHD (median = 7) did not take longer to return to school than those without ADHD (median = 7; *U* = 22,642.0, *p* = 0.81, *r* = 0.01). The survival distributions for days to return to school did not differ between students with or without ADHD (log rank: $\chi_{1}^{2}$ = 0.059, *p* = 0.81). Adolescents with ADHD were not more likely to remain out of school at 3, 5, 7, 10, 14, 21, or 28 days following injury (all *p*'s > 0.05, ORs range from 0.72 to 1.20; Table 3). Approximately one-third of students (ADHD = 35.2%, no ADHD = 33.7%) returned to school within 4 days of concussion, and two-thirds (ADHD = 64.8%, no ADHD = 67.5%) returned within 10 days. Few students had not returned fully to school by 28 days after injury (ADHD = 6.8% not returned, no ADHD = 5.7% not returned).

**Table 3:** χ^2^ Analyses comparing the percentage of students with ADHD and without ADHD who returned to school and sports at various time points after injury.

	**ADHD (*n* = 88)**	**No ADHD (*n* = 523)**			
	**%**	**%**	**χ^**2**^**	***p***	**OR (95% CI)**
**Return to school**					
3 days	29.5	27.3	0.18	0.67	0.90 (0.55–1.48)
5 days	43.2	40.7	0.19	0.67	0.90 (0.57–1.43)
7 days	54.5	55.3	0.02	0.90	1.03 (0.65–1.62)
10 days	64.8	67.5	0.25	0.62	1.13 (0.70–1.81)
14 days	83.0	77.8	1.18	0.28	0.72 (0.40–1.30)
21 days	90.9	88.7	0.37	0.54	0.79 (0.36–1.71)
28 days	93.2	94.3	0.16	0.69	1.20 (0.49–2.98)
	**ADHD (*****n*** **=** **86)**	**No ADHD (*****n*** **=** **502)**			
	**%**	**%**	***χ***^**2**^	***p***	**OR (95% CI)**
**Return to sports**					
7 days	11.6	11.4	0.01	0.94	0.97 (0.48–1.99)
10 days	31.4	25.7	1.22	0.27	0.76 (0.46–1.24)
14 days	53.5	49.4	0.49	0.48	0.85 (0.54–1.34)
21 days	80.2	75.9	0.77	0.38	0.78 (0.44–1.37)
28 days	87.2	86.1	0.08	0.77	0.91 (0.46–1.79)

*ADHD, attention-deficit/hyperactivity disorder; OR, odds ratio (values <1.0 indicate that a greater percentage of students with ADHD had returned by that time point); CI, confidence interval*.

### Return to Sports

**Figure 2:**
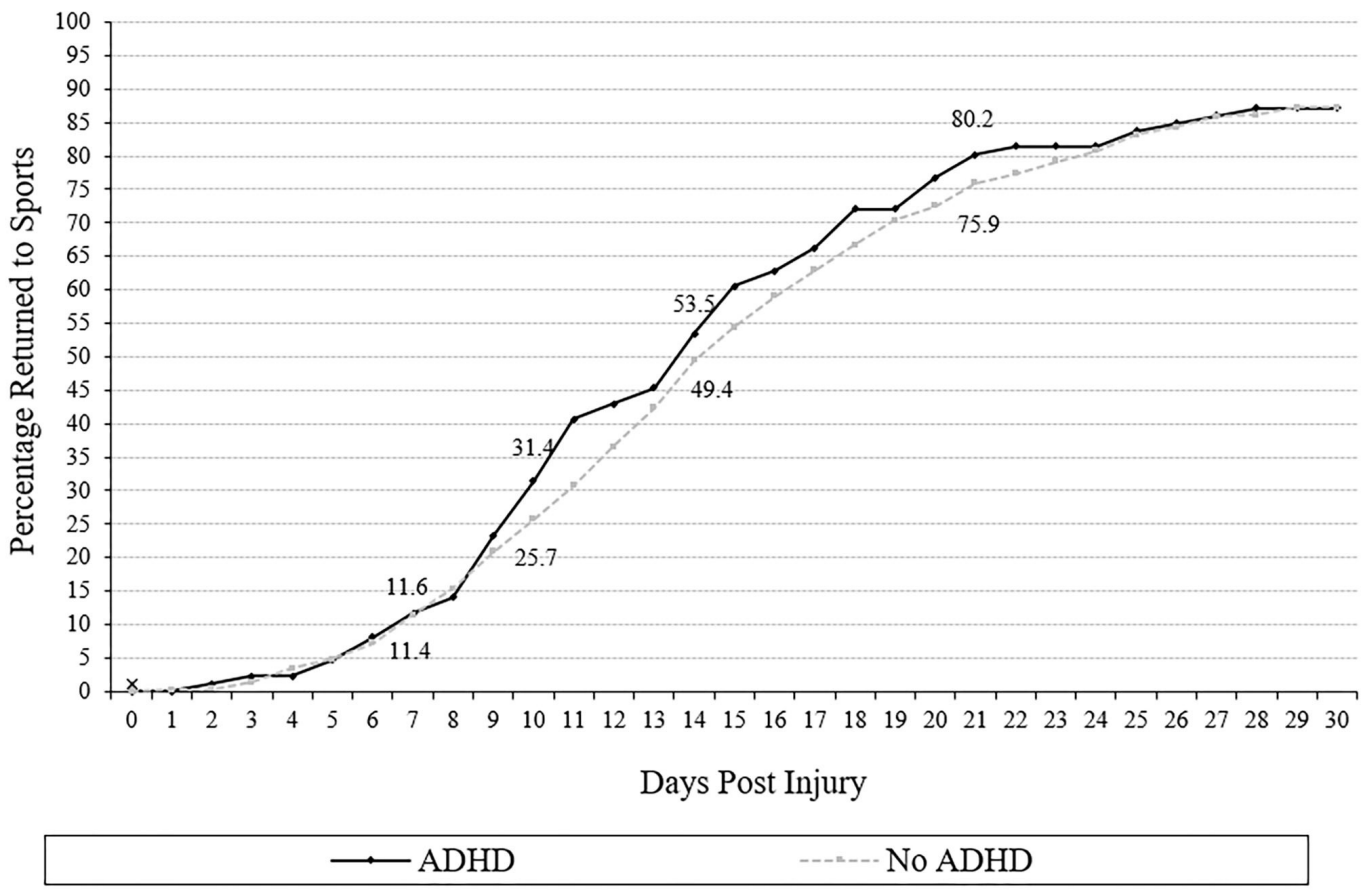
Days to return to sports (cumulative percentage curves). Cumulative percentage curves by group of time to return to sports. The featured percentages are as follows: 7 days (11.6% of those with ADHD, 11.4% of those without), 10 days (31.4% of those with ADHD, 25.7% of those without ADHD), 14 days (53.5% of those with ADHD, 49.4% of those without), and 21 days post injury (80.2% of those with ADHD, 75.9% of those without ADHD).

Cumulative recovery curves displaying the proportion of adolescents with and without ADHD who returned to sports over time are presented in Figure 2. Adolescents with ADHD (median = 14) did not take significantly longer to return to sports than those without ADHD (median = 15; *U* = 20,295.0, *p* = 0.38, *r* = 0.04). The survival distributions for days to return to sports did not differ between those with and without ADHD (log rank: $\chi_{1}^{2}$ = 0.511, *p* = 0.48). Adolescents with ADHD were not more likely to remain out of sports at 7, 10, 14, 21, or 28 days following injury (all *p*'s > 0.05, ORs range from 0.76 to 0.97; Table 3). Nearly all students (ADHD = 88.4%, no ADHD = 88.6%) took more than 7 days to return to sports. Approximately half of the adolescents (ADHD = 53.5%, no ADHD = 49.4%) returned to sports within 14 days of concussion. Between 13 and 14% of students had not returned to sports by 28 days after injury (ADHD = 12.8% not returned, no ADHD = 13.9% not returned).

### Use of Medication and Recovery Time for Adolescents With ADHD

Adolescents with ADHD who were not taking medication (median = 6) did not take significantly longer to return to school than those with ADHD who reported taking medication (median = 8; *U* = 860.5, *p* = 0.52, *r* = 0.07). Adolescents with ADHD who were not taking medications (median = 14) did not take longer to return to sports than those with ADHD who reported taking medication (median = 14; *U* = 851.0, *p* = 0.77, *r* = 0.03). Rates of adolescents with ADHD who remained out of school at 3, 5, 7, 10, 14, 21, or 28 days and sports at 7, 10, 14, 21, or 28 days following injury did not differ between those who did and did not report taking ADHD-medication (all *p*'s > 0.05).

### Concussion History and Recovery Time for Adolescents With ADHD

Adolescents with ADHD and a history of prior concussion (median = 6.5) did not take longer to return to school than those with ADHD and no history of prior concussion (median = 7; *U* = 815.5, *p* = 0.31, *r* = 0.11). Adolescents with ADHD and a history of prior concussion (median = 13) did not take longer to return to sports than those with ADHD and no history of prior concussions (median = 14; *U* = 822.5, *p* = 0.54, *r* = 0.07). Rates of adolescents with ADHD who remained out of school at 3, 5, 7, 10, or 14 days following injury did not differ between those with and without a history of prior concussion (*p*'s > 0.05). By 21 days following injury, all students with ADHD and prior concussions (100%) had returned to school, compared to 84.6% of students with ADHD and no prior concussions at 21 days ($\chi_{1}^{2}$ = 6.09, *p* = 0.01) and 88.5% of these students at 28 days ($\chi_{1}^{2}$ = 4.46, *p* = 0.04). Rates of adolescents with ADHD who remained out of sports at 7, 10, 14, 21, or 28 days following injury did not differ between those who did and did not report a history of prior concussion (all *p*'s > 0.05).

## Discussion

This is the first prospective study of concussion recovery specifically designed to examine whether ADHD is associated with worse outcome (vs. examining ADHD in secondary analyses), the largest study to date examining whether youth with ADHD are at risk of worse outcome following sport-related concussion and the first to examine days to return to school as an outcome. We leveraged data from a statewide injury surveillance platform that was integrated into school-sponsored athletics to examine 90 adolescents with ADHD. There were no differences between adolescents with ADHD and those without ADHD in days to return to school full time without accommodations or days to return to sports following concussion. Nearly all available studies have also found no statistically significant association between ADHD and worse clinical outcome from concussion, although many were hampered methodologically by small or very small sample sizes (23, 28–38), typically fewer than 15 ADHD cases (22). The two prior studies that found a significant association between ADHD and longer concussion recovery both included samples drawn from specialty concussion clinics (39, 40), which likely represent a select subgroup of adolescents who sustain concussion and are referred for specialty care. The current sample was drawn from school-based athletics using a statewide concussion surveillance platform. Further, among the two prior studies with positive findings, one included only eight youth with ADHD (39), and the other included 13 youth with ADHD (40). The current study included 90 adolescents with ADHD.

This study is also novel in that it is the first to examine whether youth with “treated” vs. “untreated” ADHD experience different concussion recovery. Among adolescents with ADHD, recovery times did not differ based on whether students reported taking medication to treat their ADHD. Additionally, youth with ADHD were more likely than those without ADHD to report having sustained a prior concussion, which is consistent with multiple prior studies (12–16). However, few prior studies have directly investigated whether prior concussions are associated with concussion recovery among those with ADHD. Concussion history is an important covariate because youth with ADHD report a greater lifetime history of concussion (13–15), and there is mixed evidence that those with prior concussions are at risk of worse clinical outcomes (21). In this study, adolescents with ADHD and prior concussions did *not* take longer to recover compared to those with ADHD and no prior concussions.

This study includes a comprehensive investigation of functional recovery, investigating how long it took for adolescents to return to school and to return to sports. Some prior studies define recovery in terms of symptom reporting and/or medical clearance (28, 30, 31, 39, 40). Our functional outcomes may make this study more clinically relevant and may control for potential confounders related to baseline differences in concussion-like symptom reporting in adolescents with ADHD (41). Moreover, school return is a highly relevant outcome for adolescents with ADHD because they are at risk of negative academic outcomes in the absence of concussion (42) and frequently have co-occurring specific learning disorders (43, 44).

The null findings that youth with ADHD, as a group, are not more likely to take longer to recover following concussion and that ADHD medication status did not influence recovery times are clinically important. There are several potentially complicating and confounding factors for clinicians to consider when determining “recovery” following concussion for youth with ADHD. First, youth with ADHD endorse many and diverse concussion-like symptoms in their daily lives (in the absence of recent concussion), including symptoms that “overlap” with ADHD, such as difficulty concentrating, but also symptoms that are not necessarily reflective of ADHD, such as headaches, dizziness, fatigue, and light sensitivity (41, 45–50). In fact, in the absence of recent concussion, many youth with ADHD endorse symptoms across a number of domains and would meet the *International Classification of Diseases, 10th Revision* symptom criteria for postconcussional syndrome (41, 46). Second, youth with ADHD perform differently on neurocognitive (48–53) and balance (45, 52) concussion assessments in the absence of recent head injury. Third, it is relatively common for youth with ADHD to present with co-occurring anxiety and depression (44), and these comorbidities are associated with concussion-like symptom reporting (46) and prolonged concussion recovery (28, 34, 38). Thus, determining recovery can be quite challenging, and clinicians are encouraged to adopt a holistic, biopsychosocial perspective when assessing and conceptualizing an adolescent's clinical status (54), especially when many weeks or months have passed since the concussion and clinicians are attempting to determine whether or the extent to which ongoing symptoms are associated with the concussion and/or other factors.

### Limitations

The health history information, including ADHD status, ADHD medication status, and concussion history, was self-reported. We were not able to verify the accuracy of adolescent self-reported health history, nor did we have details about their diagnoses, such as by whom their ADHD was diagnosed (e.g., their general practitioner/pediatrician, psychiatrist, psychologist, etc.) or medication treatment (e.g., specific medication, formulation, or dose). This limitation is common in sport concussion research. Notably, adolescent athletes asked about their health history an average of 2 years apart are highly consistent in their report of their concussion history and whether they have ADHD (55). Future studies would advance our knowledge by seeking to confirm ADHD status via methods such as parent confirmation, medical chart linkage, ADHD symptom questionnaires, and/or structured diagnostic interview, as well as collecting additional data about student athletes' ADHD diagnoses, such as age at onset and who provided their diagnosis. Moreover, these data were collected in the course of routine clinical care, primarily by athletic trainers; thus, there is likely variability in how concussions were defined/diagnosed. Similarly, there were likely differences in how some school personnel defined return to school and return to sports. There was training and ongoing technical support provided to promote data collection quality. However, some of the return-to-school data likely represented when the adolescent returned to school, not when they were back to school without accommodations. We were not able to analyze the results separately by school to determine the extent of potential variability between schools. This study examined time to return to school and sports, but data were not available regarding success or problems encountered during return or the specific accommodations and academic modifications students required or received, which represents an important area for future research. Moreover, we did not have acute post-injury assessment data to characterize the severity of all concussions. Lastly, co-occurring depression was more prevalent among those with ADHD compared to those without ADHD. We did not attempt to control for this difference because ADHD is commonly comorbid with depression (44); thus, excluding cases with comorbid depression or otherwise controlling for this covariate would have substantially decreased the representativeness of our ADHD sample.

### Conclusion

Adolescents with ADHD did not take longer to functionally recover following concussion. Among adolescents with ADHD, taking ADHD medication was not associated with recovery time or having a prior concussion history. These findings suggest that a preexisting history of ADHD, in and of itself, is not a risk factor for prolonged recovery or worse outcome following concussion. Additional research is needed to determine if there are other vulnerability factors in youth with ADHD that confer risk of worse outcome.

## Data Availability Statement

The original contributions presented in the study are included in the article, further inquiries can be directed to the corresponding author. The statistical code, syntax, output, and analyses are available to qualified researchers upon request.

## Ethics Statement

The studies involving human participants were reviewed and approved by Colby College Institutional Review Board (IRB). Written informed consent from the participants' legal guardian/next of kin was not required to participate in this study in accordance with the national legislation and the institutional requirements.

## Author Contributions

NC and GI contributed conception and design of the study. BM helped develop the injury surveillance application and organized the database. NC performed the statistical analyses and wrote the first draft of the manuscript. GI wrote sections of the manuscript. BM and PB helped design and coordinate data collection. RZ critically reviewed and edited the manuscript. PB wrote the IRB, conceptualized the overall project. All authors contributed to manuscript revision, read, and approved the submitted version.

## Conflict of Interest

GI has been reimbursed by the government, professional scientific bodies, and commercial organizations for discussing or presenting research relating to MTBI and sport-related concussion at meetings, scientific conferences, and symposiums. He has a clinical practice in forensic neuropsychology, including expert testimony, involving individuals who have sustained mild TBIs (including athletes). He has received honorariums for serving on research panels that provide scientific peer review of programs. He is a co-investigator, collaborator, or consultant on grants relating to mild TBI funded by the federal government and other organizations. He has received research support from test publishing companies in the past, including ImPACT^®^ Applications Systems, Psychological Assessment Resources, and CNS Vital Signs. He has received research support from the Harvard Integrated Program to Protect and Improve the Health of NFLPA Members, and a grant from the National Football League. He serves as a scientific advisor for BioDirection, Inc, Sway Medical, Inc., and Highmark, Inc. RZ has received salary support from the Harvard Integrated Program to Protect and Improve the Health of National Football League Players Association Members. He serves on the Scientific Advisory Board of Myomo, Oxeia Pharma, and ElMInda. The authors declare that this study received funding from the National Football League and ImPACT^®^ Applications, Inc. The funders were not involved in the study design, collection, analysis, interpretation of data, the writing of this article, or the decision to submit it for publication. The remaining authors declare that the research was conducted in the absence of any commercial or financial relationships that could be construed as a potential conflict of interest.

## References

[1] McCrory P Meeuwisse W Dvorák J Aubry M Bailes J Broglio S. Consensus statement on concussion in sport—the 5 th international conference on concussion in sport held in Berlin, October 2016. Br J Sports Med. (2017) 51:838–47. 10.1136/bjsports-2017-097699 28446457

[2] Arbogast KB Curry AE Pfeiffer MR Zonfrillo MR Haarbauer-Krupa J Breiding MJ. Point of health care entry for youth with concussion within a large pediatric care network. JAMA Pediatrics. (2016) 170:e160294. 10.1001/jamapediatrics.2016.0294 27244368 PMC6025897

[3] Itriyeva K Feinstein R Carmine L. Pediatric provider's attitudes and practices regarding concussion diagnosis and management. Int J Adolesc Med Health. (2017) 31:70. 10.1515/ijamh-2017-0070 28841573

[4] American Psychiatric Association Diagnostic and Statistical Manual of Mental Disorders. 5th ed. Arlington, VA: American Psychiatric Publishing (2013).

[5] Halstead ME Walter KD Council on Sports M, Fitness American Academy of Pediatrics. Clinical report–sport-related concussion in children and adolescents. Pediatrics. (2010) 126:597–615. 10.1542/peds.2010-2005 20805152

[6] Harmon KG Drezner JA Gammons M Guskiewicz KM Halstead M Herring SA. American medical society for sports medicine position statement: concussion in sport. Br J Sports Med. (2013) 47:15. 10.1136/bjsports-2012-091941 23243113

[7] Giza CC Kutcher JS Ashwal S Barth J Getchius TS Gioia GA. Summary of evidence-based guideline update: evaluation and management of concussion in sports: report of the guideline development subcommittee of the american academy of neurology. Neurology. (2013) 80:2250–7. 10.1212/WNL.0b013e31828d57dd 23508730 PMC3721093

[8] Lam LT. Attention deficit disorder and hospitalization due to injury among older adolescents in New South Wales, Australia. J Atten Disord. (2002) 6:77–82. 10.1177/108705470200600204 12142864

[9] Merrill RM Lyon JL Baker RK Gren LH. Attention deficit hyperactivity disorder and increased risk of injury. Adv Med Sci. (2009) 54:20–6. 10.2478/v10039-009-0022-7 19586835

[10] Pastor PN Reuben CA. Identified attention-deficit/hyperactivity disorder and medically attended, nonfatal injuries: US school-age children, 1997-2002. Ambul Pediatr. (2006) 6:38–44. 10.1016/j.ambp.2005.07.002 16443182

[11] Shilon Y Pollak Y Aran A Shaked S Gross-Tsur V. Accidental injuries are more common in children with attention deficit hyperactivity disorder compared with their non-affected siblings. Child Care Health Dev. (2012) 38:366–70. 10.1111/j.1365-2214.2011.01278.x 21722159

[12] Nelson LD Guskiewicz KM Marshall SW Hammeke T Barr W Randolph C. Multiple self-reported concussions are more prevalent in athletes with ADHD and learning disability. Clin J Sport Med. (2016) 26:120–7. 10.1097/JSM.0000000000000207 25915144 PMC6938223

[13] Iverson GL Atkins JE Zafonte R Berkner PD. Concussion history in adolescent athletes with attention-deficit hyperactivity disorder. J Neurotrauma. (2016) 33:2077–80. 10.1089/neu.2014.3424 25375785

[14] Iverson GL Wojtowicz M Brooks BL Maxwell BA Atkins JE Zafonte R. High school athletes with ADHD and learning difficulties have a greater lifetime concussion history. J Attent Disord. (2020) 24:1095–101. 10.1177/1087054716657410 27431932

[15] Iverson GL Kelshaw PM Cook NE Caswell SV. Middle school children with attention-deficit/hyperactivity disorder have a greater concussion history. Clin J Sport Med. (2020). 10.1097/JSM.0000000000000773. [Epub ahead of print]. 32032165

[16] Alosco ML Fedor AF Gunstad J. Attention deficit hyperactivity disorder as a risk factor for concussions in NCAA division-I athletes. Brain Inj. (2014) 28:472–4. 10.3109/02699052.2014.887145 24564766

[17] Anderson LE Chen ML Perrin JM Van Cleave J. Outpatient visits and medication prescribing for US children with mental health conditions. Pediatrics. (2015) 136:e1178. 10.1542/peds.2015-0807 26459647 PMC4621795

[18] Bonfield CM Lam S Lin Y Greene S. The impact of attention deficit hyperactivity disorder on recovery from mild traumatic brain injury. J Neurosurg Pediatr. (2013) 12:97. 10.3171/2013.5.PEDS12424 23905842

[19] Yeates KO Armstrong K Janusz J Taylor HG Wade S Stancin T. Long-term attention problems in children with traumatic brain injury. J Am Acad Child Adolesc Psychiatry. (2005) 44:574–84. 10.1097/01.chi.0000159947.50523.64 15908840

[20] Slomine BS Salorio CF Grados MA Vasa RA Christensen JR Gerring JP. Differences in attention, executive functioning, and memory in children with and without ADHD after severe traumatic brain injury. J Int Neuropsychol Soc. (2005) 11:645–53. 10.1017/S1355617705050769 16212692

[21] Iverson GL Gardner AJ Terry DP Ponsford JL Sills AK Broshek DK. Predictors of clinical recovery from concussion: a systematic review. Br J Sports Med. (2017) 51:941–8. 10.1136/bjsports-2017-097729 28566342 PMC5466929

[22] Cook NE Iaccarino MA Karr JE Iverson GL. Attention-deficit/hyperactivity disorder and outcome after concussion: a systematic review. J Dev Behav Pediatr. (2020) 41:571–82. 10.1097/DBP.0000000000000808 32317560

[23] Mautner K Sussman WI Axtman M Al-Farsi Y Al-Adawi S. Relationship of attention deficit hyperactivity disorder and postconcussion recovery in youth athletes. Clin J Sport Med. (2015) 25:355–60. 10.1097/JSM.0000000000000151 25353721

[24] Fritz CO Morris PE Richler JJ. Effect size estimates: current use, calculations, and interpretation. J Exp Psychol Gen. (2012) 141:2–18. 10.1037/a0024338 21823805

[25] Cohen J. Statistical power analysis for the behavioral science. 2nd ed. Hillside, NJ: Lawrence Erlbaum (1988).

[26] Chinn S. A simple method for converting an odds ratio to effect size for use in meta-analysis. Stat Med. (2000) 19:3127–31. 10.1002/1097-0258(20001130)19:22<3127::AID-SIM784>3.0.CO;2-M 11113947

[27] Cohen J. A power primer. Psychol Bull. (1992) 112:155–9. 10.1037/0033-2909.112.1.155 19565683

[28] Eisenberg MA Meehan WP III Mannix R. Duration and course of post-concussive symptoms. Pediatrics. (2014) 133:999–1006. 10.1542/peds.2014-0158 24819569 PMC4531270

[29] Ellis MJ Cordingley DM Vis S Reimer KM Leiter J Russell K. Clinical predictors of vestibulo-ocular dysfunction in pediatric sports-related concussion. J Neurosurg Pediatr. (2017) 19:38–45. 10.3171/2016.7.PEDS16310 27689244

[30] Fehr SD Nelson LD Scharer KR Traudt EA Veenstra JM Tarima SS. Risk factors for prolonged symptoms of mild traumatic brain injury: a pediatric sports concussion clinic cohort. Clin J Sport Med. (2019) 29:11–7. 10.1097/JSM.0000000000000494 29084034

[31] Hiploylee C Dufort PA Davis HS Wennberg RA Tartaglia MC Mikulis D. Longitudinal study of postconcussion syndrome: not everyone recovers. J Neurotrauma. (2017) 34:1511–23. 10.1089/neu.2016.4677 27784191 PMC5397249

[32] Lau BC Kontos AP Collins MW Mucha A Lovell MR. Which on-field signs/symptoms predict protracted recovery from sport-related concussion among high school football players? Am J Sports Med. (2011) 39:2311–8. 10.1177/0363546511410655 21712482

[33] Lau BC Collins MW Lovell MR. Cutoff scores in neurocognitive testing and symptom clusters that predict protracted recovery from concussions in high school athletes. Neurosurgery. (2012) 70:371–9; discussion:9. 10.1227/NEU.0b013e31823150f0 21841522

[34] Morgan CD Zuckerman SL Lee YM King L Beaird S Sills AK. Predictors of postconcussion syndrome after sports-related concussion in young athletes: a matched case-control study. J Neurosurg Pediatr. (2015) 15:589–98. 10.3171/2014.10.PEDS14356 25745949

[35] Nelson LD Tarima S LaRoche AA Hammeke TA Barr WB Guskiewicz K. Preinjury somatization symptoms contribute to clinical recovery after sport-related concussion. Neurology. (2016) 86:1856–63. 10.1212/WNL.0000000000002679 27164666 PMC4873681

[36] Russell K Selci E Chu S Fineblit S Ritchie L Ellis MJ. Longitudinal assessment of health-related quality of life following adolescent sports-related concussion. J Neurotrauma. (2017) 34:2147–53. 10.1089/neu.2016.4704 28077006

[37] Terwilliger VK Pratson L Vaughan CG Gioia GA. Additional post-concussion impact exposure may affect recovery in adolescent athletes. J Neurotrauma. (2016) 33:761–5. 10.1089/neu.2015.4082 26421452 PMC4840822

[38] Zemek R Barrowman N Freedman SB Gravel J Gagnon I McGahern C. Clinical risk score for persistent postconcussion symptoms among children with acute concussion in the ED. JAMA. (2016) 315:1014–25. 10.1001/jama.2016.1203 26954410

[39] Aggarwal SS Ott SD Padhye NS Meininger JC Armstrong TS. Clinical and demographic predictors of concussion resolution in adolescents: a retrospective study. Appl Neuropsychol Child. (2019) 8:50–60. 10.1080/21622965.2017.1381099 29058480

[40] Miller JH Gill C Kuhn EN Rocque BG Menendez JY O'Neill JA. Predictors of delayed recovery following pediatric sports-related concussion: a case-control study. J Neurosurg Pediatr. (2016) 17:491–6. 10.3171/2015.8.PEDS14332 26684762 PMC5476956

[41] Cook NE Sapigao RG Silverberg ND Maxwell BA Zafonte R Berkner PD. Attention-deficit/hyperactivity disorder mimics the post-concussion syndrome in adolescents. Front Pediatr. (2020) 8:2. 10.3389/fped.2020.00002 32117823 PMC7014960

[42] Arnold LE Hodgkins P Kahle J Madhoo M Kewley G. Long-term outcomes of ADHD: academic achievement and performance. J Atten Disord. (2020) 24:73–85. 10.1177/1087054714566076 25583985

[43] DuPaul GJ Gormley MJ Laracy SD. Comorbidity of LD and ADHD: implications of DSM-5 for assessment and treatment. J Learn Disabil. (2013) 46:43–51. 10.1177/0022219412464351 23144063

[44] Larson K Russ SA Kahn RS Halfon N. Patterns of comorbidity, functioning, and service use for US children with ADHD, 2007. Pediatrics. (2011) 127:462–70. 10.1542/peds.2010-0165 21300675 PMC3065146

[45] Cook NE Kelshaw PM Caswell SV Iverson GL. Children with attention-deficit/hyperactivity disorder perform differently on pediatric concussion assessment. J Pediatr. (2019) 214:168–174.e1. 10.1016/j.jpeds.2019.07.048 31477384

[46] Iverson GL Silverberg ND Mannix R Maxwell BA Atkins JE Zafonte R. Factors associated with concussion-like symptom reporting in high school athletes. JAMA Pediatr. (2015) 169:1132–40. 10.1001/jamapediatrics.2015.2374 26457403 PMC5333772

[47] Collings LJ Cook NE Porter S Kusch C Sun J Virji-Babul N. Attention-deficit/hyperactivity disorder is associated with baseline child sport concussion assessment tool third edition scores in child hockey players. Brain Inj. (2017) 31:1479–85. 10.1080/02699052.2017.1377351 28980829

[48] Cook NE Huang DS Silverberg ND Brooks BL Maxwell B Zafonte R. Baseline cognitive test performance and concussion-like symptoms among adolescent athletes with ADHD: examining differences based on medication use. Clin Neuropsychol. (2017) 31:1341–52. 10.1080/13854046.2017.1317031 28429656

[49] Brooks BL Iverson GL Atkins JE Zafonte R Berkner PD. Sex differences and self-reported attention problems during baseline concussion testing. Appl Neuropsychol Child. (2016) 5:119–26. 10.1080/21622965.2014.1003066 25923339

[50] Elbin RJ Kontos AP Kegel N Johnson E Burkhart S Schatz P. Individual and combined effects of LD and ADHD on computerized neurocognitive concussion test performance: evidence for separate norms. Arch Clin Neuropsychol. (2013) 28:476–84. 10.1093/arclin/act024 23608188

[51] Poysophon P Rao AL. Neurocognitive deficits associated with ADHD in athletes: a systematic review. Sports Health. (2018) 10:317–26. 10.1177/1941738117751387 29337649 PMC6044120

[52] Chin EY Nelson LD Barr WB McCrory P McCrea MA. Reliability and validity of the sport concussion assessment tool-3 (SCAT3) in high school and collegiate athletes. Am J Sports Med. (2016) 44:2276–85. 10.1177/0363546516648141 27281276

[53] Zuckerman SL Lee YM Odom MJ Solomon GS Sills AK Baseline neurocognitive scores in athletes with attention deficit-spectrum disorders and/or learning disability. J Neurosurg Pediatr. (2013) 12:103–9. 10.3171/2013.5.PEDS12524 23790088

[54] Iverson GL A biopsychosocial conceptualization of poor outcome from mild traumatic brain injury. In: Vasterling JJ, Bryant RA, Keane TM, editors. PTSD and Mild Traumatic Brain Injury. New York, NY: Guilford Press (2012) p. 37–60.

[55] Wojtowicz M Iverson GL Silverberg ND Mannix R Zafonte R Maxwell B. Consistency of self-reported concussion history in adolescent athletes. J Neurotrauma. (2017) 34:322–7. 10.1089/neu.2016.4412 27349296 PMC5335743

